# Future Integration of Biomarkers for Inflammatory Skin Diseases Into Daily Practice: A Survey Among a Multifaceted Group of Patients and Dermatological Care Providers

**DOI:** 10.1111/exd.70352

**Published:** 2026-09-09

**Authors:** Linda T. H. Godding, Elke M. G. J. de Jong, Sylvia van Beugen, Antoinette I. M. van Laarhoven, Els van der Pool, Nanda J. Olde, Debra Trampe, Hanna Niehues, Ilse van Ee, Annemarie Sluijmers, Robert Rissmann, Juul M. P. A. van den Reek, Marieke M. B. Seyger, the Next Generation ImmunoDermatology Consortium (NGID)

**Affiliations:** ^1^ Department of Dermatology Radboud University Medical Centre Nijmegen the Netherlands; ^2^ Institute of Psychology, Health, Medical and Neuropsychology Unit Leiden University Leiden the Netherlands; ^3^ Lectoraat Human Communication Development HAN University of Applied Sciences Nijmegen the Netherlands; ^4^ Psoriasispatiënten Nederland (PN) Nijkerk the Netherlands; ^5^ Nationale vereniging voor mensen met lupus, APS, sclerodermie en MCTD (NVLE) Utrecht the Netherlands; ^6^ Centre for Human Drug Research (CHDR) Leiden the Netherlands; ^7^ Department of Dermatology Leiden University Medical Centre Leiden the Netherlands; ^8^ Leiden Academic Centre for Drug Research (LACDR) Leiden University Leiden the Netherlands

**Keywords:** atopic dermatitis, chronic spontaneous urticaria, cutaneous lupus erythematosus, hidradenitis suppurativa, psoriasis

## Abstract

Successful implementation of dermatological biomarkers depends on early understanding of clinical needs and adoption barriers. Yet, research on these factors as perceived by dermatological care providers (DCPs) is scarce and perspectives of patients are largely underexplored. This survey study among patients and DCPs assessed biomarker needs and adoption barriers and facilitators for five inflammatory skin diseases: psoriasis (PsO), atopic dermatitis (AD), hidradenitis suppurativa (HS), cutaneous lupus erythematosus (CLE) and chronic spontaneous urticaria (CSU). In total, the study population consisted of 591 patients and 99 DCPs. Across all skin diseases, both groups mainly prioritized a biomarker for prediction of therapeutic response, however, DCPs prioritized this substantially more often than patients, especially for PsO (79.8% vs. 31.3% resp.), AD (68.7% vs. 37.1% resp.) and HS (66.7% vs. 34.4% resp.). Notably, a biomarker for the prediction of the risk to develop comorbidities was prioritized relatively often by patients (7.0%–19.4%), whereas DCPs only substantially prioritized this for CLE (14.1%). To enhance implementation, patients must ideally be able to undergo biomarker collection nearby their homes (travel time < 1 h) with complete reimbursement. For DCPs it is important that the biomarker is well validated, non‐invasive and measurable during regular visits with preferably a short sampling time.

## Graphical abstract

Across the investigated skin diseases, a therapeutic response biomarker was highly prioritized among patients and DCPs, but patients also often prioritized a biomarker for comorbidity development. Adoption barriers and facilitators differed between patients and DCPs. These differences underscore the importance to incorporate both perspectives in biomarker research and implementation efforts.

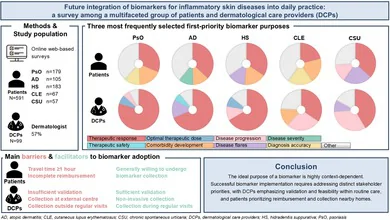

**Abbreviations**
AD: atopic dermatitis
CLE: cutaneous lupus erythematosus
CSU: chronic spontaneous urticaria
CTCL: cutaneous T‐cell lymphoma
DCP: dermatological care provider
HS: hidradenitis suppurativa
PsO: psoriasis

## 1 Introduction

The search for biomarkers in inflammatory skin diseases is extensive and has become a major focus in dermatological research [1, 2, 3, 4]. However, they have barely, if at all, found their way into everyday dermatological practice. This is mainly due to the complex road from the discovery of a potential biomarker to its final validation and implementation [5]. A biomarker can be defined as ‘any substance, structure, or process that can be measured in the body or its products and influence or predict the incidence of outcome or disease’ [6]. Biomarkers can serve multiple purposes in clinical care, including diagnosis (e.g., identifying the type of skin disease), prognosis (e.g., prediction of disease progression), monitoring (e.g., tracking disease activity over time) or assessing therapeutic success (e.g., prediction of therapeutic response). The Dutch nationwide Next Generation ImmunoDermatology (NGID)‐consortium, funded by the Dutch research council (NWO), aims to enhance personalized care by identifying biomarkers for six skin diseases: psoriasis (PsO), atopic dermatitis (AD), hidradenitis suppurativa (HS), cutaneous lupus erythematosus (CLE), chronic spontaneous urticaria (CSU) and cutaneous T‐cell lymphoma (CTCL) [7]. In line with its integrative approach, NGID also incorporates patient‐reported outcomes as potential predictors of treatment and disease outcomes, complementing biological measurements.

The need for, and priority given, to specific biomarker purposes in daily practice care can vary across different contexts. Variations may arise between diseases, due to differences in clinical presentation or treatment options, for example, as well as between patients and dermatological care providers (DCPs), due to different priorities, experiences, and expectations. In addition, patients and DCPs may perceive practical aspects of the collection and use of a biomarker as barriers or facilitators for its adoption in clinical practice [8]. Overlooking these perspectives can hinder successful implementation [9, 10, 11, 12].

While research in this field is limited, a multidisciplinary group of 35 experts, including 6 patients and 17 physicians, recently evaluated the key requirements for high‐quality biomarkers in PsO and AD, addressing both biomarker purposes and potential barriers to implementation [13]. They showed that biomarkers for the prediction of therapeutic response and disease progression were considered of main importance, and that insufficient validation would be a main obstacle to its adoption. To advance the current understanding, the present study aimed to focus on a larger and more diverse cohort of patients and DCPs in order to provide a more comprehensive understanding of biomarker needs and perceived adoption barriers and facilitators. In addition, this study broadened the scope by examining these aspects across the five inflammatory skin diseases covered by the NGID‐consortium: PsO, AD, HS, CLE and CSU. This broader focus enabled a more detailed assessment of disease‐specific needs, as well as potential differences in perspectives between the two key end‐users groups: patients and DCPs.

## 2 Methods

A cross‐sectional web‐based survey was conducted among patients with a self‐reported diagnosis of PsO, AD, HS, CLE or CSU, and among DCPs. Two separate surveys, one for patients and one for DCPs, were created using Qualtrics survey software (Qualtrics, Provo, UT) and were aligned as much as possible, with the aim of facilitating comparison across the investigated skin diseases and between the responses of patients and DCPs. Completing the survey was construed as informed consent. According to confirmation of the local Central Committee on Research Involving Human Subjects, this study was not subject to the Medical Research Involving Human Subjects Act. Data were retrieved between July and December 2024. This study was reported based on the Consensus‐Based Checklist for Reporting of Survey Studies (CROSS) [14].

### 2.1 Development of Surveys

The survey items were initially based on a study by Ziehfreund et al. [13] and developed by the core team of clinical experts and epidemiologists (Authors LTHG, MMBS, JMPAR, EMGJJ). After input from communication professionals (Authors NJO, DT, EP), psychology researchers with experience in survey design and patient‐reported measures (Authors AIML, SB), a laboratory expert (Author HN), and two patient representatives (Authors IE, AS), both surveys were pre‐tested for clarity and technical issues. The patient survey was tested by six independent dermatological researchers and one patient from an outpatient clinic. The survey for DCPs was reviewed by four dermatologists from both academic and non‐academic hospitals. After minor changes, the surveys for patients and DCPs were finalized.

### 2.2 Content

Both surveys had three main themes: (1) *Demographics*, (2) *Biomarker needs*, and (3) *Key Barriers and Facilitators* (Figure S1). The patient survey contained an additional theme assessing disease‐related demographics. Biomarker needs were assessed based on different biomarker purposes (Table S1). In the patient survey, the biomarker purposes were described using clear, patient‐friendly language rather than the technical definitions shown in Table S1. Barriers and facilitators were assessed by presenting different scenarios related to the biomarker use in daily practice, including, for example, different timings and methods of collection (Table S2). Given the early stages of biomarker development, where the frequency of collection is still undefined and the biomarker purpose can vary, patients and DCPs were instructed to answer the questions on barriers and facilitators bearing in mind the idealized overarching context in which the biomarker collection is only once and fulfills the respondent's prioritized biomarker purpose.

### 2.3 Survey Dissemination

The survey for patients was distributed via social media channels of all patient associations which are partners of the NGID‐consortium (see acknowledgements). Additionally, patients were actively approached at the outpatient clinic of various Dutch academic hospitals. The survey for DCPs was initially promoted in the Dutch Journal for Dermatology and Venereology (NTvDV) [15], and later on brought under attention in the newsletters of the Dutch Society for Dermatology and Venereology and the NGID‐consortium, and on the website of NGID (www.ng‐id.nl). Additionally, awareness for the surveys was raised via (personal) social media platforms and word‐of‐mouth advertising.

### 2.4 Statistical Analysis

All main analyses were descriptive in nature, with results presented by absolute numbers with associated percentage, mean ± standard deviation (SD) or median with associated range. Biomarker needs were analysed according to skin disease. First, the prioritized biomarker purposes by patients and DCPs were assessed. Additionally, a sensitivity analysis was performed according to the patients' self‐reported satisfaction with their state of skin disease. For this analysis, patients were dichotomized into dissatisfied patients and satisfied patients, based on their responses on the 5‐point Likert scale regarding satisfaction with state of skin disease ((very) dissatisfied resp. (very) satisfied). In addition to the main descriptive analyses, exploratory, hypothesis‐generating univariable binary logistic regression analyses were performed to assess the associations between the baseline demographic and clinical characteristics of patients and biomarker prioritization. Analyses were restricted to the most frequently prioritized biomarker purposes by patients. Results are presented as odds ratios (ORs) with 95% confidence intervals (CIs) and corresponding nominal *p*‐values (two‐sided *p* < 0.05). No correction for multiple testing was applied. This was the only inferential statistical analysis performed in the study. The patterns of the top three ranking of biomarker purposes by patients were illustrated by Sankey diagrams. Data on key barriers and facilitators were analysed separately for the total populations of patients and DCPs. Data were analysed using SPSS version 29.0 (IBM Corp, Armonk, NY, USA). Figures were created with Microsoft Excel (version 2408) and Rstudio version 2024.04.02.

## 3 Results

### 3.1 Study Population

In total, 781 patients and 128 DCPs responded to the survey. Respondents were required to complete the subtheme on biomarker needs, as this was the primary focus of this study. Responses were marked as incomplete if this section was left unfinished, which was the case for 124 patients and 29 DCPs (Figure S1). Additionally, patients were excluded if they reported a skin disease outside the scope of this study, did not specify their type of skin disease, had multiple eligible skin diseases or were under the age of 18. The final study sample consisted of 591 patients and 99 DCPs.

Most patient responders had HS (*N* = 183; 31.0%), PsO (*N* = 179; 30.3%) or AD (*N* = 105; 17.8%) (Table 1a). The majority was female (83.6%) with a median age of 49 years [18.0–80.0]. The median disease duration of the total patient population was 18 years [0–72.0], with substantial variation seen across the skin diseases. Among the 99 DCPs, most were female (67.7%) with a median age of 37 years [26.0–68.0] (Table 1b). The predominant profession was dermatologist (56.6%). DCPs were employed in academic (63.6%) and/or in non‐academic hospitals (38.4%).

**TABLE 1:** Demographics at time of survey completion (a) patients; (b) DCPs.

(a) Patients	Total (*n* = 591)	PsO (*n* = 179)	AD (*n* = 105)	HS (*n* = 183)	CLE (*n* = 67)	CSU (*n* = 57)
Gender, *n* (%)						
Male	95 (16.1)	52 (29.1)	31 (29.5)	3 (1.6)	5 (7.5)	4 (7.0)
Female	494 (83.6)	126 (70.4)	74 (70.5)	180 (98.4)	62 (92.5)	52 (91.2)
Other/unknown	2 (0.3)	1 (0.6)	—	—	—	1 (1.8)
Age, years, median [range]	49.0	58.0	49.0	41.0	55.0	49.0
	[18–80]	[18–80]	[18–80]	[18–69]	[19–78]	[21–71]
Disease duration, years, median [range]	18.0	24.0	39.0	10.0	20.0	6.0
	[0–72]	[0–69]	[1–72]	[0–38]	[045]	[0–26]
Treatment type, *n* (%)^a^, ^b^						
None	56 (9.5)	15 (8.4)	4 (3.8)	31 (16.9)	4 (6.0)	2 (3.5)
Topical	232 (39.3)	67 (37.4)	63 (60.0)	61 (33.3)	36 (53.7)	5 (8.8)
Phototherapy	23 (3.9)	17 (9.5)	2 (1.9)	3 (1.6)	—	1 (1.8)
Systemic	225 (38.1)	60 (33.5)	53 (50.5)	54 (29.5)	25 (37.3)	33 (57.9)
Surgical	14 (2.4)	—	—	14 (7.7)	—	—
Other^c^	12 (2.0)	3 (1.7)	3 (2.9)	5 (2.7)	1 (1.5)	—
Missing	171 (28.9)	57 (31.8)	18 (17.1)	56 (30.6)	21 (31.3)	20 (35.1)
Satisfaction status skin disease, *n* (%)^a^						
Very dissatisfied	53 (9.0)	19 (10.6)	9 (8.6)	17 (9.3)	3 (4.5)	5 (8.8)
Dissatisfied	128 (21.7)	37 (20.7)	21 (20.0)	48 (26.2)	13 (19.4)	9 (15.8)
Neutral	83 (14.0)	16 (8.9)	13 (12.4)	30 (16.4)	16 (23.9)	8 (14.0)
Satisfied	111 (18.8)	32 (17.9)	30 (28.6)	30 (16.4)	9 (13.4)	10 (17.5)
Very satisfied	45 (7.6)	19 (10.6)	14 (13.3)	2 (1.1)	5 (7.5)	5 (8.8)
Missing	171 (28.9)	56 (31.3)	18 (17.1)	56 (30.6)	21 (31.3)	20 (35.1)

(b) DCPs	Total (*N* = 99)
Gender, *n* (%)	
Male	31 (31.3)
Female	67 (67.7)
Other/Unknown	—
Age, years, median [range]	37.0 [26–68]
Job title, *n* (%)	
Dermatologist	56 (56.6)
Resident in‐training	24 (24.2)
Resident not in‐training	8 (8.1)
Medical PhD student providing dermatological care	10 (10.1)
Other	2 (2.0)
Workplace, *n* (%)^b^	
Academic hospital	63 (63.6)
Non‐academic hospital	38 (38.4)
Independent treatment center	6 (6.1)
Other	2 (2.0)

Abbreviations: AD, atopic dermatitis; CLE, cutaneous lupus erythematosus; CSU, chronic spontaneous urticaria; DCPs, dermatological care providers; HS, hidradenitis suppurativa; PhD, doctor of philosophy; PsO, psoriasis.

^a^ Filled in by a subtotal of 420 patients (see Figure S1), including 123 PsO, 87 AD, 127 HS, 46 CLE and 37 CSU patients.

^b^ Multiple answers were allowed.

^c^ For example, homeopathic medication, dietary regimens or supplements.

### 3.2 Biomarker Needs

**FIGURE 1:**
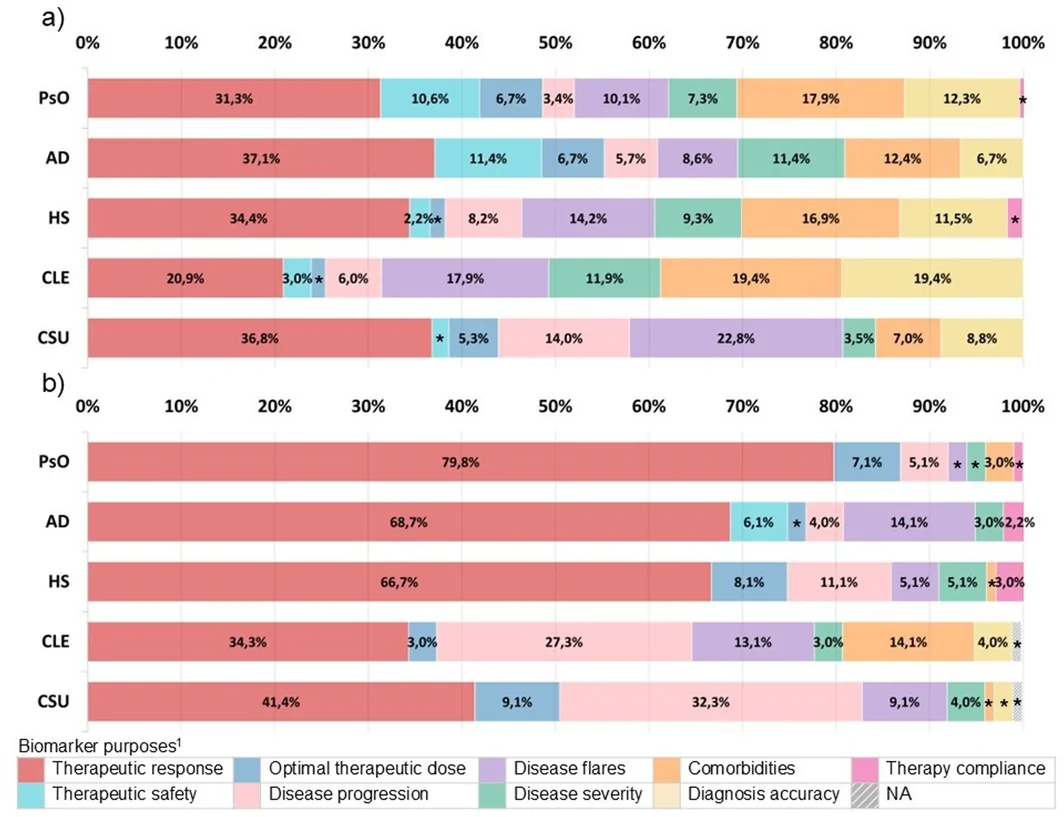
Prioritized biomarker purpose by patients and DCPs, according to skin disease. (a) Patients; (b) DCPs. *Represent a bar between 0.1% and 2%. ^1^Definitions of biomarker purposes shown in Table S1. AD, atopic dermatitis; CLE, cutaneous lupus erythematosus; CSU, chronic spontaneous urticaria; DCP, dermatological care provider; HS, hidradenitis suppurativa; NA, not applicable; PsO, psoriasis.

Patients generally prioritized a greater variety of biomarker purposes than DCPs (Figure 1). Interestingly, both patients and DCPs most often prioritized the biomarker purpose ‘therapeutic response’ across all skin diseases, but DCPs prioritized this substantially more often than patients, in particular for PsO (79.8%), AD (68.7%) and HS (66.7%). Patients also frequently prioritized the biomarker purpose ‘comorbidities’ (7.0%–19.4%), even second most frequently prioritized among those with PsO, AD, HS and CLE. In contrast, DCPs only substantially (14.1%) prioritized the biomarker purpose ‘comorbidities’ for CLE. While across all skin diseases a substantial proportion of patients prioritized the biomarker purpose ‘diagnosis accuracy’ (8.8%–19.4%), DCPs rarely prioritized this biomarker purpose (0%–4.0%). Patients with CLE and CSU second most often prioritized the biomarker purpose ‘disease flares’, whereas DCPs second most often prioritized the biomarker purpose ‘disease progression’ for these skin conditions.

Figure S2 provides a clear visualization of the direction and magnitude of the differences in prioritized biomarker purposes between patients and DCPs, further reinforcing the patterns observed in the main analysis. For the biomarker purpose ‘therapeutic response’, percentage‐point differences were consistently skewed towards DCPs, reaching up to 48.5 percentage points in PsO. In contrast, differences for ‘comorbidities’ and ‘diagnosis accuracy’ were, across all skin diseases, consistently skewed towards patients. For PsO, AD and HS, the largest discrepancies between patients and DCPs were observed for the biomarker purposes ‘therapeutic response’ and ‘comorbidities’. DCPs prioritized ‘therapeutic response’ more frequently than patients, with differences of 48.5 percentage points in PsO, 31.6 in AD and 32.3 in HS. Patients prioritized ‘comorbidities’ more frequently, with corresponding differences of 14.9, 12.4 and 15.9 percentage points. For both CLE and CSU, the largest DCP‐favoring gap was seen for ‘disease progression’ (21.3 and 18.3 percentage points, respectively). The largest patient‐favoring gap differed by disease, with ‘diagnosis accuracy’ showing the largest gap in CLE (15.4 percentage points), whereas ‘disease flares’ showed the largest gap in CSU (13.7 percentage points).

**FIGURE 2:**
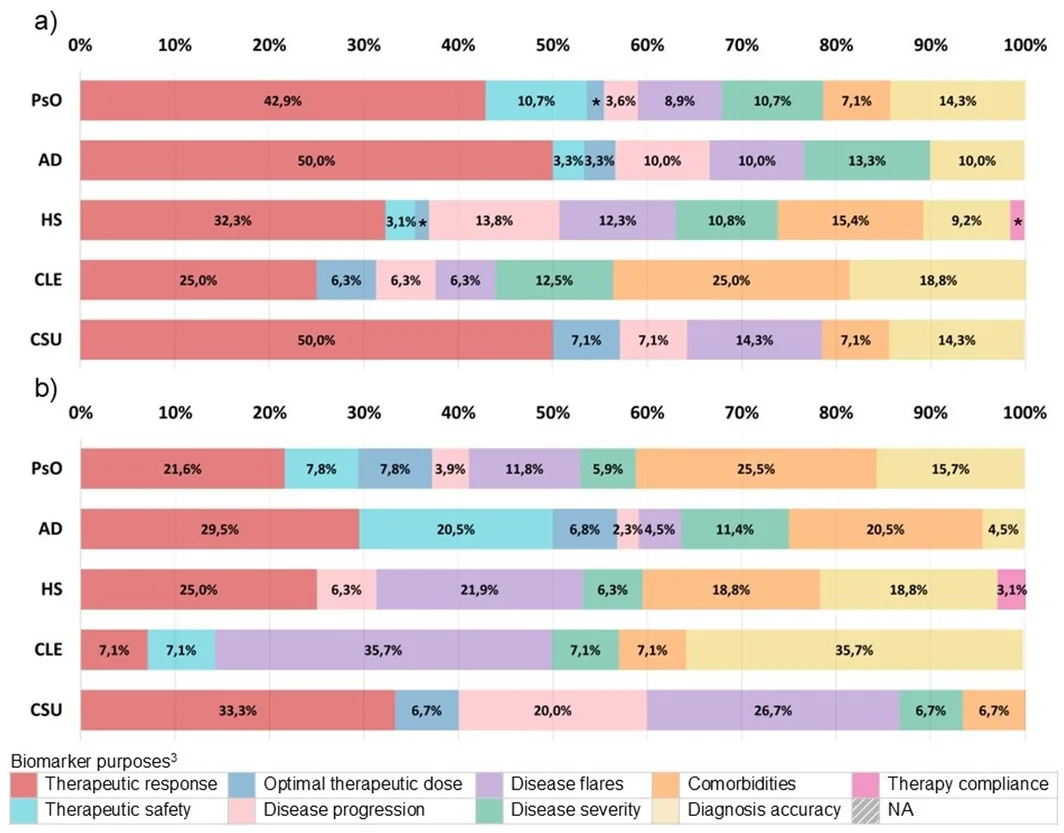
Prioritized biomarker purpose by patients according to skin disease, stratified by satisfaction with state of skin disease. (a) Dissatisfied patients^1^; (b) Satisfied patients^2^. *Represent a bar between 0.1% and 2% ^1^Patients were classified as dissatisfied when they reported to be (very) dissatisfied on the 5‐point Likert scale within the theme on disease‐related demographics. ^2^Patients were classified as satisfied when they reported to be (very) satisfied on the 5‐point Likert scale within the theme on disease‐related demographics. ^3^Definitions of biomarker purposes shown in Table S1. AD, atopic dermatitis; CLE, cutaneous lupus erythematosus; CSU, chronic spontaneous urticaria; HS, hidradenitis suppurativa; PsO, psoriasis.

The prioritized biomarker purpose by patients according to their self‐reported satisfaction with their state of skin disease, revealed notable differences across groups (Figure 2). Overall, the biomarker purpose ‘therapeutic response’ was more frequently prioritized by patients who were dissatisfied with their disease status (25.0%–50.0%) compared to those who were satisfied (7.1%–33.3%). For PsO, the exploratory, univariable binary logistic regression analysis (Table S3a) similarly indicated an association between satisfaction with disease status and a lower odds of prioritizing ‘therapeutic response’ (OR: 0.37; 95% CI: 0.16–0.86). Compared to dissatisfied patients, satisfied patients with PsO and AD more frequently prioritized the biomarker purpose ‘comorbidities’ (25.5% resp. 20.5%). The exploratory binary logistic regression analysis yielded a similar pattern in patients with PsO, showing that satisfaction with disease status was associated with a higher likelihood of prioritizing the biomarker purpose ‘comorbidities’ (OR: 4.45; 95% CI: 1.35–14.71). For patients with AD, no reliable estimate could be obtained, as none of the dissatisfied patients prioritized the biomarker purpose ‘comorbidities’. Compared to dissatisfied patients, satisfied patients with HS, CLE and CSU identified the biomarker purpose ‘disease flares’ as particularly important (21.9% resp. 35.7% resp. 26.7%).

An additional file shows the detailed assessment and patterns of the top 3 biomarker purposes of patients according to skin disease (see Figure S3). In addition, the proportion of patients with a biomarker purpose in their top 3 is shown (see Table S4). These findings demonstrate additional diversity in prioritized biomarker purposes across skin diseases which was not fully captured within the primary analysis (Figure 1). The biomarker purpose ‘therapeutic safety’ was most frequently ranked within the top three by patients with AD (41.9%) and least frequently by patients with HS (12.6%). Similarly, the ranking of the biomarker purpose ‘optimal therapeutic dose’ varied substantially between groups, with only 11.9% of patients with CLE including it in their top three, compared to 36.8% of patients with CSU. In addition, the selection of ‘disease severity’ among the top three biomarker purposes differed more clearly across conditions compared to the primary analysis, with approximately 30.0% of patients with HS and CLE ranking it in their top three, whereas this proportion was considerably lower among patients with CSU (8.8%).

### 3.3 Key Barriers & Facilitators

Over 90% of the patients would be willing to undergo a single biomarker collection in daily practice regardless of the moment of biomarker collection, the person who collects the biomarker, the method of biomarker collection and whether the biomarker is commonly known among other patients and DCPs (Table 2). Patients expressed most reluctance towards a travel time of more than 1 h (39.7%) and incomplete reimbursement of the biomarker (29.6%). Notably, DCPs (Table 3) held differing views: the vast majority of DCPs (91.4%) would be encouraged to collect the biomarker during regular visits, whereas collection outside regular visits was a barrier to most DCPs (52.7%). With regard to the method of biomarker collection, it was notable that the majority of patients expressed (complete) willingness to undergo a biopsy or blood sampling. DCPs were more neutral towards a biopsy or blood sampling and especially encouraged with a non‐invasive collection procedure. Interestingly, all DCPs (100%) would be encouraged to adopt a biomarker if it is well validated, whereas validation seemed to be less influential for patients.

**TABLE 2:** Key barriers and facilitators perceived by patients.^a^

	Completely unwilling (%)	Unwilling (%)	Total unwilling (%)	Neutral (%)	Willing (%)	Completely willing (%)	Total willing (%)
Moment of biomarker collection							
Outside regular visits	2.1	6.9	9.0	6.2	24.5	60.3	84.8
During regular visits	0.9	0.9	1.8	4.4	12.2	81.7	93.9
Location of biomarker collection							
At external center (< 1 h travel time)	1.8	4.4	6.2	3.2	21.1	69.5	90.6
At external center (≥ 1 h travel time)	14.0	25.7	39.7	8.9	25.9	25.5	51.4
At internal center	0.5	1.4	1.9	3.7	12.8	81.7	94.5
Person responsible for biomarker collection							
Treating physician	0.7	0.7	1.4	5.7	10.8	82.1	92.9
Assistant	3.2	6.2	9.4	5.5	20.4	64.7	85.1
Patient	1.8	3.9	5.7	4.4	12.6	77.3	89.9
Method of biomarker collection							
Blood sampling	0.7	2.3	3.0	2.1	13.1	81.9	95.0
Biopsy	3.4	8.5	11.9	4.4	27.3	56.4	83.7
Questionnaire	0.5	1.8	2.3	2.5	13.5	81.7	95.2
Non‐invasive	0.9	2.3	3.2	3.7	10.8	82.3	93.1
Biomarker knowledge							
Commonly known	0.9	2.5	3.6	9.2	19.3	68.1	87.4
Not commonly known	1.8	6.7	8.5	10.3	32.6	48.6	81.2
Biomarker validation							
Well validated	0.9	0.7	1.6	4.8	10.8	82.8	93.6
Not well validated	2.5	8.7	11.2	9.6	32.1	47.0	79.1
Costs for society							
High	4.1	13.3	17.4	25.0	25.2	32.3	57.5
Low	0.7	1.8	2.5	14.4	9.2	73.9	83.1
Reimbursement							
Complete	0.5	1.1	1.6	5.0	9.2	84.2	93.4
Incomplete	14.2	15.4	29.6	9.9	30.0	30.5	60.5

*Note:* The results shown above reflect data from 436 patients (73.8% of the total sample of patients).

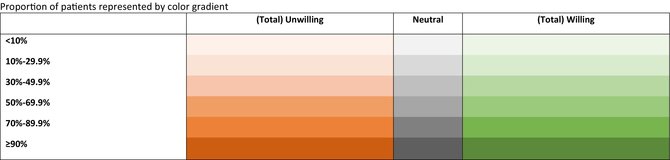

^a^ All hypothetical situations were presented in a specific context; considering a one‐time biomarker collection and that the biomarkers' purpose aligns with the respondent's highest priority.

**TABLE 3:** Key barriers and facilitators perceived by DCPs.^a^

	Very discouraged (%)	Discouraged (%)	Total discouraged (%)	Neutral (%)	Encouraged (%)	Very encouraged (%)	Total encouraged (%)
Moment of biomarker collection							
Outside regular visits	10.8	41.9	52.7	43.0	2.2	2.2	4.4
During regular visits	—	1.1	1.1	7.5	59.1	32.3	91.4
Location of biomarker collection							
At external center	16.1	53.8	69.9	26.9	3.2	—	3.2
At internal center	—	2.2	2.2	14.0	53.8	30.1	83.9
Person responsible for biomarker collection							
Treating physician	2.2	22.6	24.8	67.7	4.3	3.2	7.5
Assistant	—	1.1	1.1	11.8	50.5	36.6	87.1
Patient	—	1.1	1.1	20.4	43.0	35.5	78.5
Method of biomarker collection							
Blood sampling	1.1	3.2	4.3	50.5	35.5	9.7	45.2
Biopsy	2.2	17.2	19.4	61.3	17.2	2.2	19.4
Questionnaire	4.3	17.2	21.5	30.1	33.3	15.1	48.4
Non‐invasive	—	—	—	6.5	46.2	47.3	93.5
Biomarker knowledge							
Commonly known	—	—	—	18.3	58.1	23.7	81.8
Not commonly known	—	34.4	34.4	58.1	5.4	2.2	7.6
Biomarker validation							
Well validated	—	—	—	—	48.4	51.6	100
Not well validated	32.3	59.1	91.4	7.5	—	1.1	1.1
Biomarker costs							
≤ Biopsy	—	2.2	2.2	18.3	55.9	23.7	79.6
> Biopsy	3.2	39.8	43.0	53.8	3.2	—	3.2
Biomarker analysis							
At external center	9.7	41.9	51.6	46.2	2.2	—	2.2
At internal center	—	—	—	12.9	59.1	28.0	87.1
Guideline availability	1.1	1.1	2.2	15.1	60.2	22.6	82.8

*Note:* The results shown above reflect data from 93 DCPs (93.9% of the total sample of DCPs).

Abbreviation: DCPs, dermatological care providers.

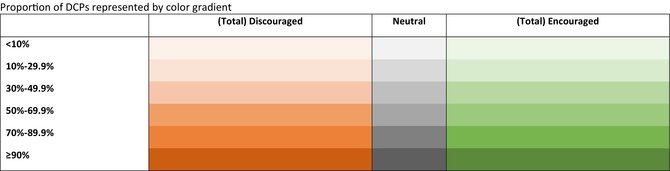

^a^ All hypothetical situations were presented in a specific context; considering a one‐time biomarker collection and that the biomarkers' purpose aligns with the respondent's highest priority.

### 3.4 Acceptable Time Investment for Biomarker Collection

**FIGURE 3:**
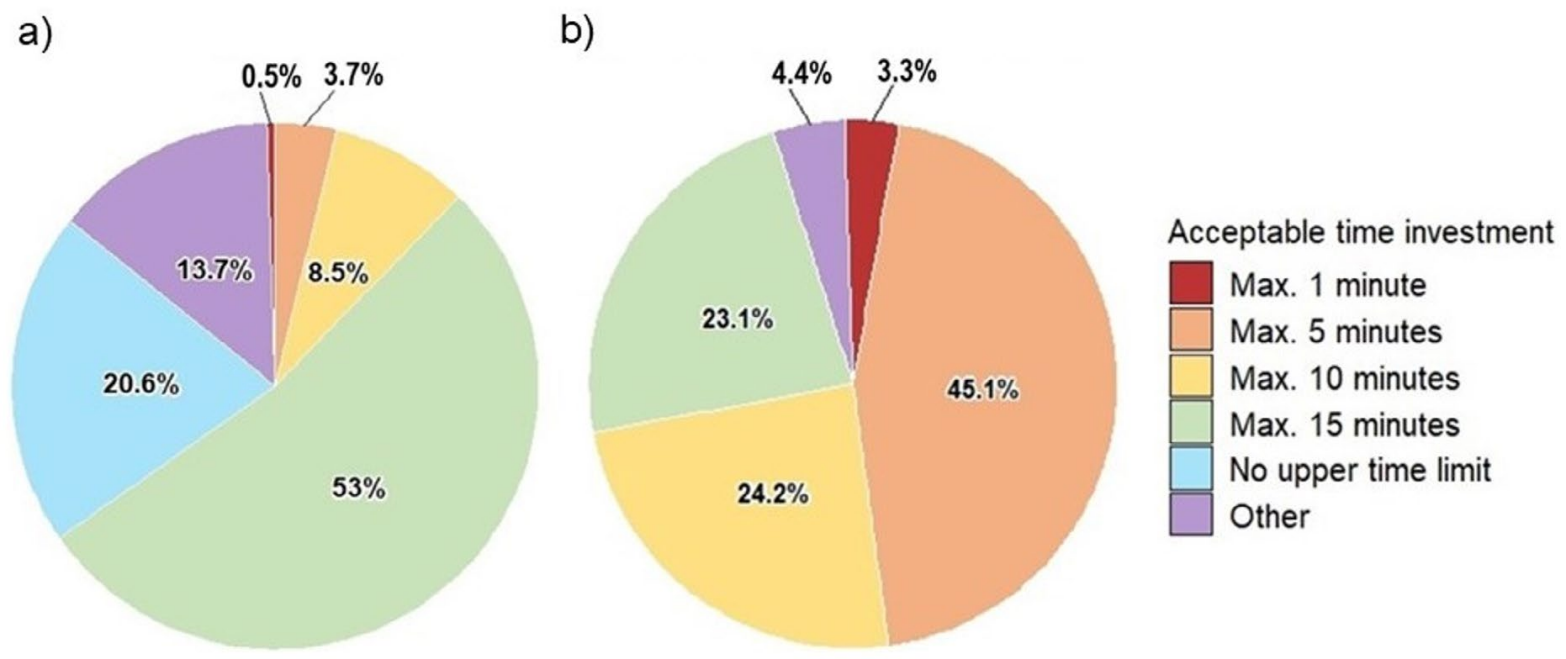
Acceptable time investment of biomarker collection by patients and DCPs. (a) Patients; (b) DCPs. DCPs, dermatological care providers.

The majority (53.0%) of patients reported that 15 min would be an acceptable time investment for biomarker collection, and a substantial proportion (20.6%) expressed no upper time limit (Figure 3). In contrast, the majority (45.1%) of DCPs reported a maximum acceptable time investment of 5 min, and none of the DCPs were willing to accept an unlimited time investment.

## 4 Discussion

This study presents the biomarker needs of a large group of patients (*N* = 591) and DCPs (*N* = 99) along with the perceived adoption barriers and facilitators regarding five inflammatory skin diseases: PsO, AD, HS, CLE and CSU. While both groups primarily emphasized a predictor for therapeutic response across the skin diseases, this need was particularly prominent among DCPs for PsO (79.8%), AD (68.7%) and HS (66.7%). Remarkably, patients more often (7.0%–19.4%) prioritized a biomarker capable of predicting the risk to develop comorbidities compared with DCPs (0%–14.1%). A long travel time (≥ 1 h) and incomplete reimbursement were considered as main adoption challenges by patients, whereas for DCPs, insufficient validation clearly posed the main adoption barrier.

Although direct comparison with a previous study on high‐quality biomarkers for PsO and AD is not possible due to the fact that they had a Delphi study design and therefore a smaller sample size (patients: *n* = 6; healthcare providers: *n* = 17), our findings similarly indicate the need for a biomarker capable of predicting therapeutic response among patients and DCPs [13]. However, the unique comparison between patients and DCPs in this study revealed that this need is predominantly expressed by DCPs, particularly for PsO (79.8%), whereas the needs of patients were more diverse, with priorities across several domains. It is a remarkable finding that such a high proportion of DCPs prioritized therapeutic response for PsO, given the availability of multiple highly efficacious biologics as treatment options compared to the other investigated skin diseases. The expressed need may reflect the increasing challenge DCPs face in choosing the most appropriate treatment for each patient from a broad range of biologics with different working mechanisms. A biomarker for therapeutic response could facilitate in the selection of the optimal treatment and improve person‐centered care.

Patients across all skin diseases more often prioritized a biomarker capable of predicting the risk to develop comorbidities compared to DCPs. Interestingly, this need especially stood out among patients with PsO and AD who expressed satisfaction with their state of skin disease at the time of survey completion (25.5% resp. 20.5%). This finding may suggest that, when patients perceive their therapeutic response as adequate, attention shifts towards the importance of, among others, comorbidities. Remarkably, DCPs did not often prioritize a biomarker for comorbidities, despite the accumulating evidence of the development of comorbidities in the investigated inflammatory skin diseases [16, 17, 18, 19, 20], and the discussion in the literature about the possible importance of early adequate treatment to prevent comorbidities [21, 22, 23]. We observed that only for CLE, a substantial proportion of DCPs (14.1%) prioritized this biomarker, which may be attributed to concerns regarding the potential progression of CLE to systemic lupus erythematosus (SLE). Exploratory univariable binary logistic regression analyses (Table S3) also suggested an association between satisfaction with disease status and prioritization of the biomarker purpose ‘comorbidities’ for PsO. Although the results of these analyses should be interpreted with caution due to small subgroup sizes and the lack of adjustment for potential confounders, some other potentially informative patterns were observed. This may indicate that biomarker priorities differ between patient subgroups. Future studies with larger study populations are needed to confirm these observations.

For CLE and CSU, it was observed that a substantial proportion of DCPs prioritized a predictive biomarker for disease progression (27.3% resp. 32.3%), while interest in such a biomarker for PsO, AD and HS was markedly lower (4.0%–11.1%). It may be hypothesized that this need arises from the heterogeneous disease courses of patients with CLE and CSU. Patients, however, did not often prioritize a biomarker for disease progression across all skin diseases (3.6%–13.8%). The difference in findings between patients and DCPs illustrates the value of including the patient voice in shaping future biomarker research agendas.

Another finding was that, across all skin diseases, a substantial proportion of patients (6.7%–19.4%) prioritized a biomarker for diagnostic accuracy, while this seemed no priority for DCPs (except for CLE, where DCPs prioritized it in 4%). This difference may be explained by the fact that DCPs are highly trained in the recognition and diagnosis of the investigated skin diseases whereas patients often had already undergone a prolonged diagnostic trajectory, starting with the general practitioner before being referred to a DCP. These findings may suggest that patients value a more rapid diagnosis than currently achieved timelines in daily clinical practice.

By describing the biomarker needs among patients and DCPs, this study provides insight into priorities for future research on purpose‐specific biomarkers. Currently, the field is rapidly expanding, with several candidate biomarkers reported within the literature across the five investigated skin diseases. The literature reflects a broad spectrum of biomarker applications, ranging from diagnostic and monitoring biomarkers [1, 24], to predictive biomarkers for disease progression [3, 25] and therapeutic response [26, 27, 28, 29, 30]. While some biomarkers have been studied extensively, such as HLA‐C*06:02 in relation to treatment response in patients with PsO [26], the majority of studies remain at an early stage with limited translation into clinical practice. This contrasts with, for example, dermato‐oncology, where biomarkers such as tumour thickness for patient survival [31] and BRAF mutation status in advanced‐stage melanoma to guide treatment decisions [32] are already well established. A consistently highlighted limitation in the biomarker literature for inflammatory skin diseases is that, despite the identification of numerous candidate biomarkers, most remain insufficiently validated for routine clinical use [1, 25, 26, 27]. Validation is already recognized as a requirement for high‐quality biomarkers in both PsO and AD [13], and the present study's finding that DCPs perceive sufficient validation as the main facilitator to biomarker adoption underscores its essential role. The present findings on biomarker needs provide guidance for how future validation efforts should be prioritized.

Beyond validation, time‐related factors emerged as important facilitators among DCPs, such as collection during regular patient visits and a short sampling time. This corresponds with findings from other medical fields, indicating that the adoption of procedures demanding substantial time can pose significant barriers to successful implementation [33, 34, 35]. Future research should focus on barriers and facilitators in a context that the biomarker needs to be collected repeatedly, as this might influence the perceived time‐related barriers and facilitators.

Overall, patients would be well willing to undergo biomarker collection within the specific context that the collection is only once and provides the patient with information related to their prioritized biomarker purpose. Interestingly, the majority of patients (80%) indicated willingness to undergo a biopsy for biomarker collection. DCPs, on the other hand, considered a non‐invasive biomarker collection as one of the most important facilitators. To better understand the perspectives of both patients and DCPS towards skin biopsies as biomarker collection method, further research is required.

A major strength of this study was the focus on perspectives from both patients and DCPs, thereby enabling a detailed assessment of different perspectives. Additionally, the patient population was diverse with regard to age, disease duration and satisfaction with state of skin disease. Despite its strengths, the study is subject to some limitations. Although efforts were made to enhance the understanding of the topic on biomarkers, certain drawbacks should be acknowledged. First, despite the elaborate explanation of a biomarker and its intended purposes within the surveys, some semantic variations in interpretation may have occurred. Secondly, the complexity of the subject may have reduced patients' willingness to participate. This might have resulted in a selection bias towards patients who are more educated, possibly within the medical domain. In addition, it might have been that patients or DCPs with little confidence in biomarkers hesitated to fill in the survey. Lastly, the study relied on self‐reported diagnoses provided by patients, rather than confirmed diagnoses by the physician. Note that every survey has a responder bias which is inherent to this methodology.

While both patients and DCPs often prioritized a biomarker for prediction of therapeutic response, with DCPs exhibiting the strongest preference, patients notably diverge from DCPs by prioritizing a broader range of biomarker purposes, often prioritizing a biomarker that predicts the risk to develop comorbidities. Identifying a relevant biomarker for inflammatory skin diseases requires careful consideration of the specific disease subtype as well as the individual patient profile. For future successful implementation, patients prefer to undergo the biomarker collection nearby their homes and with complete reimbursement. For DCPs, it is important that the biomarker is well validated, and can be collected non‐invasively, during regular patient visits, with preferably a short sampling time. The strong emphasis on the patients' perspective and the comparison with the DCPs' point of view on biomarkers provides novel and valuable insights that can inform future biomarker research in inflammatory skin diseases. It should be emphasized that, even when biomarkers are analytically and clinically valid, those developed primarily from a clinician‐driven perspective risk limited real‐world implementation if they fail to address patients' needs. This underscores the need to incorporate the patient perspective throughout the biomarker development process. The priority gaps identified between patients and DCPs represent an important first step towards this goal, and towards the successful implementation of biomarkers in clinical practice more broadly. Future qualitative research is required to assess the reasoning behind biomarker needs and perceived adoption barriers and facilitators by patients and DCPs.

## Author Contributions

L.T.H.G.: conceptualization, methodology, formal analysis, investigation, writing – original draft, visualization. E.M.G.J.J., J.M.P.A.R. and M.M.B.S.: conceptualization, methodology, investigation, writing – original draft, supervision. S.B., A.I.M.L., E.P., N.J.O., D.T., H.N., I.E., A.S.: methodology, investigation, writing – review and editing. R.R.: investigation, writing – review and editing.

## Funding

This study was funded by the Dutch Research Council (NWO) NWA‐ORC project NWA.1389.20.182 entitled Next Generation ImmunoDermatology (NGID).

## Ethics Statement

According to confirmation of the independent local Central Committee on Research Involving Human Subjects (Research Ethics Committee Radboud University Nijmegen Medical Centre), this study was not subject to the Medical Research Involving Human Subjects Act (ref. 2024–17 177).

## Consent

According to Dutch law, informed consent was not mandatory for this noninterventional study. All respondents gave informed consent for participation before starting the survey.

## Conflicts of Interest

L.T.H.G. has received a speaking fee from Almirall, LEO Pharma, Eli Lilly and Sanofi. The funding is not personal but goes directly to the Department of Dermatology, Radboud University Medical Centre (Radboudumc), Nijmegen, The Netherlands. E.M.G.J.J. has received research grants for the independent research fund of the Department of Dermatology, Radboud University Medical Centre (Radboudumc), Nijmegen, The Netherlands, from AbbVie, BMS, Janssen Pharmaceutica, Leo Pharma, Novartis and UCB for research on psoriasis and has acted as a consultant and/or paid speaker for and/or participated in research sponsored by companies that manufacture drugs used for the treatment of psoriasis or eczema, including AbbVie, Amgen, Almirall, Boehringer Ingelheim, BMS, Celgene, Galapagos, Janssen Pharmaceutica, LEO Pharma, Eli Lilly, Novartis, Sanofi and UCB. All funding is not personal, but goes directly to the Department of Dermatology, Radboud University Medical Centre (Radboudumc), Nijmegen, the Netherlands. S.B. has received speaking/consulting fees from AbbVie, Benecke and Janssen. I.E. is a volunteer Patient representative for Psoriasis Patiënten Nederland and serves as an IFPA Ambassador. In this capacity, they bring the patient perspective to various projects focused on life with psoriasis and the care for people living with psoriasis or asked as a speaker. Some of these projects receive funding from pharmaceutical companies, including AbbVie, Janssen, Novartis, BMS, Almirall, UCB, Boehringer Ingelheim, LEO Pharma, All funding is provided to the respective organizations (Psoriasis Patiënten Nederland and IFPA), and not received personally. J.M.P.A.R. carried out clinical trials for AbbVie, Celgene, Almirall and Janssen and has received speaking fees/attended advisory boards from AbbVie, Janssen, Zoetis, BMS, Almirall, LEO Pharma, Novartis, UCB and Eli Lilly and reimbursement for attending or chairing a symposium from Janssen, Pfizer, Celgene and AbbVie. All funding is not personal but goes to the independent research fund of the department of dermatology of Radboudumc Nijmegen, the Netherlands. M.M.B.S. received grants from/was involved in clinical trials with AbbVie, Amgen, Celgene, Eli Lilly, Janssen, LEO Pharma and Pfizer. She served as a consultant for AbbVie, Eli Lilly, Janssen, Leo Pharma, Novartis, Pfizer and UCB; fees were paid directly to the Department of Dermatology, Radboud University Medical Centre (Radboudumc), Nijmegen, The Netherlands. No other disclosures were reported.

## Supporting information


**Table S1:** Definitions of biomarker purposes.
**Table S2:** Hypothetical situations presented to patients and DCPs for the assessment of key barriers and facilitators.*
**Table S3:** Exploratory, hypothesis‐generating univariable binary logistic regression output for the association between demographic and clinical characteristics and biomarker prioritization, according to skin disease.
**Table S4:** Proportion of patients including a biomarker purpose in their top 3.
**Figure S1:** Schematic overview of respondent selection and survey structure.
**Figure S2:** Diverging bar charts presenting differences between prioritized biomarker purposes between patients and DCPs, according to skin disease.
**Figure S3:** Top 3 ranking of biomarker purposes according to type of skin disease.

## Data Availability

The data that support the findings of this study are available from the corresponding author upon reasonable request.
