# Supplementary material for: Future Integration of Biomarkers for Inflammatory Skin Diseases Into Daily Practice: A Survey Among a Multifaceted Group of Patients and Dermatological Care Providers
Source: Exp Dermatol. 2026 Sep 9;35(9):e70352. doi: 10.1111/exd.70352 (PMC13557849; doi:10.1111/exd.70352)
Supplement: Supplementary file 1 [file EXD-35-e70352-s001.docx]

Supplementary material

Table S1. Definitions of biomarker purposes

| Biomarker purpose | Definition |
| --- | --- |
| Therapeutic response | The biomarker predicts the therapeutic response, for efficacy maximization |
| Therapeutic safety | The biomarker predicts the risk to develop side-effects from a certain treatment, for toxicity minimization |
| Optimal therapeutic dose | The biomarker predicts which dose of a treatment is the most optimal for a patient, both in terms of effectiveness and safety |
| Disease progression | The biomarker predicts disease progression |
| Disease flares | The biomarker predicts if and/or when disease flares will occur |
| Disease severity | The biomarker accurately reflects the disease severity |
| Comorbidities | The biomarker predicts the risk to develop a comorbidity (e.g. allergic asthma in patients with AD or psoriatic arthritis in patients with PsO) |
| Diagnosis accuracy | The biomarker accurately reflects the diagnosis and differentiates between types of skin diseases |
| Therapy compliance | The biomarker reflects whether the patient adheres to the treatment as prescribed |

AD, atopic dermatitis; PsO, psoriasis

Table S2. Hypothetical situations presented to patients and DCPs for the assessment of key barriers and facilitators*

| **Hypothetical situations** | | **Presented to** | |
| --- | --- | --- | --- |
|  |  | **Patients** | **DCPs** |
| **Moment of biomarker collection**  outside regular visits  during regular visits | The biomarker collection…  …has to be done outside the regular planned visits of patients.  …can be done during the regular planned visits of patients. | X  X | X  X |
| **Location of biomarker collection**  at external centre  at external centre (travel time < 1 hour)  at external centre (travel time ≥1 hour)  at internal centre | The biomarker collection…  …has to be done at an external centre, other than where the patient is treated (no travel time indicated).  …has to be done at an external centre, other than where the patient is treated. The travel time is < 1 hour.  …has to be done at an external centre, other than where the patient is treated. The travel time is ≥ 1 hour.  …can be done at the centre where the patient is treated. | X  X  X | X  X |
| **The person who collects the biomarker**  Treating physician  Physician-assistant  Patient | The biomarker collection…  …has to be done by the treating physician.  …can be done by the physician-assistant.  …can be done by the patient. | X  X  X | X  X  X |
| **The method of biomarker collection**  Blood sampling  Biopsy  Questionnaire  Non-invasive | The biomarker collection is…  …by blood sampling.  …by biopsy.  …by questionnaire(s).  …a non-invasive procedure. | X  X  X  X | X  X  X  X |
| **Biomarker analysis**  at external centre  at internal centre | The biomarker analysis…  …has to be done at an external centre (other than collection).  …can be done at the centre where the collection takes place. |  | X  X |
| **Biomarker knowledge**  Commonly known  Not commonly known | Other DCPs or patients are aware of its existence.  Other DCPs or patients are unaware of its existence. | X  X | X  X |
| **Biomarker validation**  Well validated  Not well validated | The biomarker…  …is well validated.  …is not well validated. | X  X | X  X |
| **Guideline availability** | There is a guideline available on how and when to use the biomarker. |  | X |
| **Biomarker costs**  ≤ than biopsy  > than biopsy | The costs associated with the biomarker are…  …equal to or lower than those of a biopsy.  …higher than those of a biopsy. |  | X  X |
| **Costs for society**  High  Low | The costs for society are high.  The costs for society are low. | X  X |  |
| **Reimbursement**  Complete  Incomplete | The biomarker…  …is completely reimbursed.  …is not completely reimbursed. | X  X |  |

^*^All hypothetical situations were presented in a specific context; considering a one-time biomarker collection and that the biomarkers’ purpose aligns with the respondent’s highest priority

DCP, dermatological care provider

Figure S1. Schematic overview of respondent selection and survey structure

| **Patients**  Total sample of respondents (**N=781**) | **DCPs**  Total sample of respondents (**N=128**) |
| --- | --- |

| Exclusion due to:   - Incomplete response^1^(**N=124**) - Duplicate response (**N=3**) - Non-eligible/unknown skin disease (**N=52**) - Multiple skin diseases (**N=6**) - Age <18 years (**N=5**) | Exclusion due to:   - Incomplete response^1^ (**N=29**) |
| --- | --- |

| Total sample of patients (**N=591**) | Total sample of DCPs (**N=99**) |
| --- | --- |

| **Demographics** | |
| --- | --- |
| (*Completed by* *100%*)  Sex, year of birth, type of skin disease | (*Completed by 100%*)  Sex, year of birth, profession, workplace |

| **Biomarker needs** | |
| --- | --- |
| (*Completed by 100%*)   1. Selection of biomarker purposes^2^ according to needs (multiple choice) 2. Ranking previously chosen biomarker purposes^2^ in order of importance^3^ | (*Completed by 100%*)   1. Ranking biomarker purposes^2^ in order of importance for the investigated skin diseases as a group 2. Prioritization of one biomarker purpose^2^ per investigated skin disease |

| **Key Barriers and facilitators** | |
| --- | --- |
| (*Completed by 73.7%*)   1. Rate willingness to undergo biomarker collection in presented hypothetical situations^4^ (5-point Likert scale)   (*Completed by 71.4%*)   2. Reporting perceived maximum acceptable time investment for biomarker collection | (*Completed by 93.9%*)   1. Rate encouragement to use a biomarker in daily practice in presented hypothetical situations^4^ (5-point Likert scale)   (*Completed by 91.0%*)   2. Reporting perceived maximum acceptable time investment for biomarker collection |

| **Disease-related demographics** |  |
| --- | --- |
| (*Completed by 70.9%)*  Year of diagnosis, current treatment, satisfaction with state of skin disease (5-Point Likert Scale) |  |

DCP, dermatological care provider

^1^Incomplete responses were defined as respondents who did not complete the survey theme *Biomarker needs*

^2^The list of biomarker purposes and the definitions is shown in Table S1

^3^Variation in the total number of biomarker purposes ranked across patients corresponded to differences in their selected options in the multiple-choice question.

^4^The presented hypothetical situations are stated in Table S2

Figure S2. Diverging bar charts presenting differences between prioritized biomarker purposes between patients and DCPs, according to skin disease

*Note. The ordering of biomarker purposes differs across skin diseases and is based on the magnitude of the differences observed between DCPs and patients*

a. PsO

b. AD

c. HS

d. CLE

e. CSU

AD, atopic dermatitis; CLE, cutaneous lupus erythematosus; CSU, chronic spontaneous urticaria; DCP, dermatological care provider; HS, hidradenitis suppurativa; PsO, psoriasis

Table S3. Exploratory, hypothesis-generating univariable binary logistic regression output for the association between demographic and clinical characteristics and biomarker prioritization, according to skin disease

*Note 1. Reported p-values are nominal*

*Note 2. For some associations, no estimate could be obtained due to sparse data. If this is the case, this is indicated with NA*

a. PsO (N=179)

|  | Therapeutic response | (n=56/179)^†^ | Comorbidities | (n=32/179)^†^ | Disease flares | (n=18/179)^†^ | Diagnosis accuracy | (n=22/179)^†^ |
| --- | --- | --- | --- | --- | --- | --- | --- | --- |
| Gender  Male  Female | Ref.  **2.42 (1.11 – 5.26) P < 0.05** | (n=10/178)  (n=46/178) | Ref.  1.01 (0.43 – 2.37) P = 0.98 | (n=9/178)  (n=22/178) | Ref.  1.50 (0.47 – 4.79) P = 0.49 | (n=4/178)  (n=14/178) | Ref.  1.12 (0.41 – 3.03) P = 0.83 | (n=6/178)  (n=16/178) |
| Age | 1.00 (0.97 – 1.02) P = 0.64 | (n=56/179) | 1.00 (0.97 – 1.03) P = 0.90 | (n=32/179) | 1.00 (0.97 – 1.04) P = 0.98 | (n=18/179) | 0.99 (0.96 – 1.02) P = 0.61 | (n=22/179) |
| Disease duration | 1.00 (0.98 – 1.02) P = 0.86 | (n=56/179) | 0.99 (0.96 – 1.02) P = 0.55 | (n=32/179) | 1.02 (0.98 – 1.05) P = 0.32 | (n=18/179) | 1.00 (0.97 – 1.02) P = 0.71 | (n=22/179) |
| Treatment type  Non-systemic treatment  Systemic treatment | Ref.  **0.42 (0.19 – 0.92) P < 0.05** | (n=26/122)  (n=14/122) | Ref.  2.17 (0.80 – 5.90) P = 0.13 | (n=7/122)  (n=13/122) | Ref.  1.04 (0.32 – 3.42) P = 0.95 | (n=6/122)  (n=6/122) | Ref.  1.32 (0.51 – 3.46) P = 0.57 | (n=9/122)  (n=11/122) |
| Satisfaction status skin disease  Dissatisfied  Neutral  Satisfied | Ref.  0.80 (0.26 – 2.51) P = 0.70  **0.37 (0.16 – 0.86) P < 0.05** | (n=24/123)  (n=6/123)  (n=11/123) | Ref.  3.00 (0.60 – 15.09) P = 0.18  **4.45 (1.35 – 14.71) P< 0.05** | (n=4/123)  (n=3/123)  (n=13/123) | Ref.  0.68 (0.07 – 6.28) P = 0.73  1.36 (0.39 – 4.76) P = 0.63 | (n=5/123)  (n=1/123)  (n=6/123) | Ref.  2.00 (0.52 – 7.77) P = 0.32  1.11 (0.39 – 3.23) P = 0.84 | (n=8/123)  (n=4/123)  (n=8/123) |

Number of participants per demographic or clinical characteristic: Gender 178 (Male 52, Female 126); Age 179; Disease duration 179; Treatment type 122 (Non-systemic treatment 62, systemic treatment 60); Satisfaction status skin disease 123 (Dissatisfied 56, Neutral 16, Satisfied 51)

^†^Number of participants who prioritized the respective biomarker purpose/Total number of participants with respective disease. Note that data on *treatment type* and *satisfaction status skin disease* were missing for some participants; therefore, the numbers do not add up to the total N of participants who prioritized the respective biomarker purpose and the total number of participants included in the model is lower.

b. AD (N=105)

|  | Therapeutic response | (n=39/105)^†^ | Comorbidities | (n=13/105)^†^ | Disease flares | (n=9/105)^†^ | Diagnosis accuracy | (n=7/105)^†^ |
| --- | --- | --- | --- | --- | --- | --- | --- | --- |
| Gender  Male  Female | Ref.  **3.35 (1.23 – 9.13) P < 0.05** | (n=6/105)  (n=33/105) | Ref.  1.46 (0.37 – 5.71) P = 0.59 | (n=3/105)  (n=10/105) | Ref.  0.82 (0.19 – 3.52) P = 0.79 | (n=3/105)  (n=6/105) | Ref.  0.53 (0.11 – 2.54) P = 0.43 | (n=3/105)  (n=4/105) |
| Age | 0.99 (0.97 – 1.06) P = 0.47 | (n=39/105) | 1.03 (0.99 – 1.07) P = 0.18 | (n=13/105) | 0.97 (0.93 – 1.01) P = 0.25 | (n=9/105) | 1.06 (1.00 – 1.13) P = 0.05 | (n=7/105) |
| Disease duration | 1.01 (0.99 – 1.03) P = 0.46 | (n=39/105) | 0.99 (0.96 – 1.02) P = 0.65 | (n=13/105) | 1.00 (0.96 – 1.04) P = 0.96 | (n=9/105) | 1.01 (0.96 – 1.06) P = 0.69 | (n=7/105) |
| Treatment type  Non-systemic treatment  Systemic treatment | Ref.  0.68 (0.28 – 1.65) P = 0.39 | (n=14/87)  (n=17/87) | Ref.  0.88 (0.26 – 3.04) P = 0.84 | (n=5/87)  (n=7/87) | Ref.  1.08 (0.24 – 4.83) P = 0.92 | (n=3/87)  (n=5/87) | Ref.  0.96 (0.15 – 6.07) P = 0.97 | (n=2/87)  (n=3/87) |
| Satisfaction status skin disease  Dissatisfied  Neutral  Satisfied | Ref.  0.30 (0.07 – 1.31) P = 0.11  0.42 (0.16 – 1.10) P = 0.08 | (n=15/87)  (n=3/87)  (n=13/87) | Ref.  NA  NA | (n=0/87)  (n=3/87)  (n=9/87) | Ref.  2.70 (0.47 – 15.65) P = 0.27  0.43 (0.07 – 2.74) P = 0.57 | (n=3/87)  (n=3/87)  (n=2/87) | Ref.  NA  0.43 (0.07 – 2.74) P = 0.37 | (n=3/87)  (n=0/87)  (n=2/87) |

Number of participants per demographic or clinical characteristic: Gender 105 (Male 31, Female 74); Age 105; Disease duration 105; Treatment type 87 (Non-systemic treatment 34, systemic treatment 53); Satisfaction status skin disease 87 (Dissatisfied 30, Neutral 13, Satisfied 44)

^†^Number of participants who prioritized the respective biomarker purpose/Total number of participants with respective disease. Note that data on *treatment type* and *satisfaction status skin disease* were missing for some participants; therefore, the numbers do not add up to the total N of participants who prioritized the respective biomarker purpose and the total number of participants included in the model is lower.

c. HS (N=183)

|  | Therapeutic response | (n=63/183)^†^ | Comorbidities | (n=31/183)^†^ | Disease flares | (n=26/183)^†^ | Diagnosis accuracy | (n=21/183)^†^ |
| --- | --- | --- | --- | --- | --- | --- | --- | --- |
| Gender  Male  Female | Ref.  1.05 (0.09 – 11.82) P = 0.97 | (n=1/183)  (n=62/183) | Ref.  0.40 (0.04 – 4.55) P = 0.46 | (n=1/183)  (n=30/183) | Ref.  NA | (n=0/183)  (n=26/183) | Ref.  NA | (n=0/183)  (n=21/183) |
| Age | 0.99 (0.96 – 1.02) P = 0.53 | (n=63/183) | 1.00 (0.96 – 1.03) P = 0.82 | (n=31/183) | 1.01 (0.97 – 1.05) P = 0.61 | (n=26/183) | 1.01 (0.97 – 1.05) P = 0.63 | (n=21/183) |
| Disease duration | 1.02 (0.99 – 1.06) P = 0.20 | (n=63/183) | 1.00 (0.96 – 1.05) P = 0.87 | (n=31/183) | 0.97 (0.92 – 1.02) P = 0.28 | (n=26/183) | 0.99 (0.94 – 1.04) P = 0.57 | (n=21/183) |
| Treatment type  Non-systemic treatment  Systemic treatment | Ref.  1.67 (0.78 – 3.58) P = 0.19 | (n=19/127)  (n=20/127) | Ref.  1.28 (0.20 – 3.28) P = 0.61 | (n=11/127)  (n=10/127) | Ref.  1.43 (0.55 – 3.73) P = 0.46 | (n=10/127)  (n=10/127) | Ref.  0.43 (0.15 – 1.28) P = 0.13 | (n=14/127)  (n=5/127) |
| Satisfaction status skin disease  Dissatisfied  Neutral  Satisfied | Ref.  1.04 (0.42 – 2.63) P = 0.92  0.70 (0.27 – 1.81) P = 0.46 | (n=21/127)  (n=10/127)  (n=18/127) | Ref.  1.10 (0.34 – 3.56) P = 0.87  1.27 (0.42 – 3.87) P = 0.68 | (n=10/127)  (n=5/127)  (n=6/127) | Ref.  1.43 (0.42 – 4.79) P = 0.58  2.00 (0.65 – 6.10) P = 0.23 | (n=8/127)  (n=5/127)  (n=7/127) | Ref.  2.99 (0.91 – 9.86) P = 0.07  2.27 (0.67 – 7.70) P = 0.19 | (n=6/127)  (n=7/127)  (n=6/127) |

Number of participants per demographic or clinical characteristic: Gender 183 (Male 3, Female 180); Age 183; Disease duration 183; Treatment type 127 (Non-systemic treatment 73, systemic treatment 54); Satisfaction status skin disease 127 (Dissatisfied 65, Neutral 30, Satisfied 32)

^†^Number of participants who prioritized the respective biomarker purpose/Total number of participants with respective disease. Note that data on *treatment type* and *satisfaction status skin disease* were missing for some participants; therefore, the numbers do not add up to the total N of participants who prioritized the respective biomarker purpose and the total number of participants included in the model is lower.

d. CLE (N=67)

|  | Therapeutic response | (n=14/67)^†^ | Comorbidities | (n=13/67)^†^ | Disease flares | (n=12/67)^†^ | Diagnosis accuracy | (n=13/67)^†^ |
| --- | --- | --- | --- | --- | --- | --- | --- | --- |
| Gender  Male  Female | Ref.  NA | (n=0/67)  (n=14/67) | Ref.  0.34 (0.05 – 2.17) P = 0.25 | (n=2/67)  (n=11/67) | Ref.  NA | (n=0/67)  (n=12/67) | Ref.  0.96 (0.10 – 9.39) P = 0.97 | (n=1/67)  (n=12/67) |
| Age | 1.00 (0.96 – 1.05) P = 0.90 | (n=14/67) | 1.01 (0.97 – 1.06) P = 0.56 | (n=13/67) | 0.98 (0.94 – 1.03) P = 0.44 | (n=12/67) | 1.00 (0.96 – 1.05) P = 0.90 | (n=13/67) |
| Disease duration | 0.98 (0.92 – 1.05) P = 0.52 | (n=14/67) | 1.01 (0.96 – 1.07) P = 0.69 | (n=13/67) | 0.98 (0.92 – 1.04) P = 0.54 | (n=12/67) | 0.99 (0.94 – 1.04) P = 0.71 | (n=13/67) |
| Treatment type  Non-systemic treatment  Systemic treatment | Ref.  1.14 (0.23 – 6.00) P = 0.87 | (n=3/46)  (n=4/46) | Ref.  1.06 (0.25 – 4.60) P = 0.94 | (n=4/46)  (n=5/46) | Ref.  0.81 (0.18 – 3.83) P = 0.79 | (n=4/46)  (n=4/46) | Ref.  0.63 (0.16 – 2.44) P = 0.50 | (n=6/46)  (n=5/46) |
| Satisfaction status skin disease  Dissatisfied  Neutral  Satisfied | Ref.  0.43 (0.07 – 2.77) P = 0.37  0.23 (0.02 – 2.37) P = 0.22 | (n=4/46)  (n=2/46)  (n=1/46) | Ref.  1.00 (0.20 – 4.96) P = 1.00  0.23 (0.02 – 2.37) P = 0.22 | (n=4/46)  (n=4/46)  (n=1/46) | Ref.  2.14 (0.17 – 26.33) P = 0.55  8.33 (0.84 – 83.17) P = 0.07 | (n=1/46)  (n=2/46)  (n=5/46) | Ref.  1.00 (0.17 – 5.90) P = 1.00  2.41 (0.46 – 12.72) P = 0.30 | (n=3/46)  (n=3/46)  (n=5/46) |

Number of participants per demographic or clinical characteristic: Gender 67 (Male 5, Female 62); Age 67; Disease duration 67; Treatment type 46 (Non-systemic treatment 21, systemic treatment 25);

Satisfaction status skin disease 46 (Dissatisfied 16, Neutral 16, Satisfied 14)

^†^Number of participants who prioritized the respective biomarker purpose/Total number of participants with respective disease. Note that data on *treatment type* and *satisfaction status skin disease* were missing for some participants; therefore, the numbers do not add up to the total N of participants who prioritized the respective biomarker purpose and the total number of participants included in the model is lower.

e. CSU (N=57)

|  | Therapeutic response | (n=21/57)^†^ | Comorbidities | (n=4/57)^†^ | Disease flares | (n=13/57)^†^ | Diagnosis accuracy | (n=5/57)^†^ |
| --- | --- | --- | --- | --- | --- | --- | --- | --- |
| Gender  Male  Female | Ref.  NA | (n=4/56)  (n=16/56) | Ref.  NA | (n=0/56)  (n=4/56) | Ref.  NA | (n=0/56)  (n=13/56) | Ref.  NA | (n=0/56)  (n=5/56) |
| Age | 1.01 (0.96 – 1.05) P = 0.73 | (n=21/57) | 0.98 (0.90 – 1.06) P = 0.59 | (n=4/57) | 1.05 (0.99 – 1.11) P = 0.10 | (n=13/57) | 1.02 (0.95 – 1.10) P = 0.59 | (n=5/57) |
| Disease duration | 0.95 (0.86 – 1.04) P = 0.27 | (n=21/57) | **1.16 (1.01 – 1.33) P < 0.05** | (n=4/57) | 0.88 (0.72 – 1.07) P = 0.18 | (n=13/57) | 1.09 (0.97 – 1.24) P = 0.16 | (n=5/57) |
| Treatment type  Non-systemic treatment  Systemic treatment | Ref.  2.21 (0.21 – 23.56) P = 0.51 | (n=1/37)  (n=14/37) | Ref.  NA | (n=0/37)  (n=4/37) | Ref.  0.54 (0.05 – 6.24) P = 0.62 | (n=1/37)  (n=5/37) | Ref.  NA | (n=0/37)  (n=4/37) |
| Satisfaction status skin disease  Dissatisfied  Neutral  Satisfied | Ref.  0.60 (0.10 – 3.54) P = 0.57  0.50 (0.11 – 2.24) P = 0.37 | (n=7/37)  (n=3/37)  (n=5/37) | Ref.  4.33 (0.33 – 57.65) P = 0.27  0.93 (0.05 – 16.42) P = 0.96 | (n=1/37)  (n=2/37)  (n=1/37) | Ref.  NA  2.18 (0.33 – 14.36) P = 0.42 | (n=2/37)  (n=0/37)  (n=4/37) | Ref.  2.00 (0.22 – 17.89) P = 0.54  NA | (n=2/37)  (n=2/37)  (n=0/37) |

Number of participants per demographic or clinical characteristic: Gender 56 (Male 4, Female 52); Age 57; Disease duration 57; Treatment type 37 (Non-systemic treatment 4, systemic treatment 33);

Satisfaction status skin disease 37 (Dissatisfied 14, Neutral 8, Satisfied 15)

^†^Number of participants who prioritized the respective biomarker purpose/Total number of participants with respective disease. Note that data on *treatment type* and *satisfaction status skin disease* were missing for some participants; therefore, the numbers do not add up to the total N of participants who prioritized the respective biomarker purpose and the total number of participants included in the model is lower.

AD, atopic dermatitis; CLE, cutaneous lupus erythematosus; CSU, chronic spontaneous urticaria; HS, hidradenitis suppurativa; PsO, psoriasis

Figure S3. Top 3 ranking of biomarker purposes according to type of skin disease

a. PsO

b. AD

c. HS

d. CLE

e. CSU

AD, atopic dermatitis; CLE, cutaneous lupus erythematosus; CSU, chronic spontaneous urticaria; HS, hidradenitis suppurativa; PsO, psoriasis

Table S4. Proportion of patients including a biomarker purpose in their top 3

|  | **Therapeutic response** | **Therapeutic safety** | **Optimal therapeutic dose** | **Disease progression** | **Disease flares** | **Disease severity** | **Comorbidities** | **Diagnosis accuracy** | **Therapy compliance** |
| --- | --- | --- | --- | --- | --- | --- | --- | --- | --- |
| **PsO** | 55.9% | 33.0% | 29.1% | 12.8% | 28.5% | 19.0% | 40.8% | 17.3% | 2.2% |
| **AD** | 64.8% | 41.9% | 26.7% | 19.0% | 34.3% | 20.0% | 37.1% | 9.5% | 2.9% |
| **HS** | 65.6% | 12.6% | 21.9% | 33.3% | 37.2% | 28.4% | 36.6% | 17.5% | 3.8% |
| **CLE** | 52.2% | 20.9% | 11.9% | 32.8% | 37.3% | 31.3% | 38.8% | 31.3% | 1.5% |
| **CSU** | 64.9% | 26.3% | 36.8% | 28.1% | 43.9% | 8.8% | 26.3% | 12.3% | 1.8% |

AD, atopic dermatitis; CLE, cutaneous lupus erythematosus; CSU, chronic spontaneous urticaria; HS, hidradenitis suppurativa; PsO, psoriasis
